# Therapeutic alliance during trauma focused treatment in adolescent and young adult patients with PTSD

**DOI:** 10.1186/s12888-024-06410-x

**Published:** 2025-01-13

**Authors:** Anne Grass, Rita Rosner, Angelina Ciner, Babette Renneberg, Regina Steil

**Affiliations:** 1 Department of Clinical Psychology and Psychotherapy, Institute of Psychology, Goethe University Frankfurt, Varrentrappstr. 40-42, 60486 Frankfurt am Main, Germany; 2 Department of Psychology, Catholic University Eichstätt-Ingolstadt, Ostenstr. 25, 85072 Eichstätt, Germany; 3 Department of Clinical Psychology and Psychotherapy, Freie Universität Berlin, Habelschwerdter Allee 45, 14195 Berlin, Germany

**Keywords:** Alliance, PTSD, Adolescents, Abuse, Treatment outcome

## Abstract

**Background:**

Greater therapeutic alliance has been associated with an improved treatment outcome in various clinical populations. However, there is a lack of evidence for this association in posttraumatic stress disorder (PTSD) in young patients. We therefore investigated the development of the therapeutic alliance during Developmentally adapted cognitive processing therapy (D-CPT) in adolescents and young adults with PTSD following abuse to answer the question whether there was a connection between the therapeutic alliance and symptom reduction.

**Methods:**

Weekly assessments of therapeutic alliance, rated by patients and their therapists, as well as PTSD symptom severity from a randomized controlled trial (RCT) of D-CPT were analyzed with multilevel modelling. The sample consisted of *n* = 39 patients aged 14–21 with a history of sexual and/or physical abuse.

**Results:**

Therapeutic alliance increased during D-CPT. The ratings of the therapeutic alliance by patients and therapists were strongly correlated (*r* = .512, *p* < .01); however, at session level, there was a significant difference between the patients’ and their therapists’ alliance assessments. Patients with a higher perceived therapeutic alliance showed a greater reduction in self-reported symptoms over the course of therapy, compared to patients with lower alliance ratings. However, this only applied to the therapeutic alliance assessed by the patients.

**Discussion:**

The therapeutic alliance plays a crucial role in D-CPT with young patients, contributing to a reduction in symptom severity over the course of treatment. It is essential that therapists prioritize the development of a strong alliance and seek feedback from their patients. The results suggest that patients’ perceptions, which often differ from therapists’ assessments, were more important in determining treatment success in the sample. Studies with larger samples sizes and additional independent ratings of alliance are needed to further examine the alliance-outcome link.

**Trial registration:**

The trial was registered at the German Clinical Trial Registry, DRKS00004787, 18 March 2013, https://www.drks.de/DRKS00004787.

## Background

The therapeutic alliance, sometimes also referred to as working or helping alliance, originated in psychoanalysis, but is now one of the most frequently studied factors of all forms of psychotherapy [1]. The definition developed by Bordin [2] which applies equally to all therapeutic modalities, comprises primarily three factors that characterize the therapeutic alliance: First, mutual agreement on the goals of therapy (e.g. symptom reduction). Second, mutual agreement on the tasks of therapy (e.g. the therapeutic tasks during treatment to achieve the chosen goals) and third, the bond between client and therapist (e.g. trust) [2]. In addition, Hatcher & Barends [3] emphasized the dyadic nature of the therapeutic alliance. When the therapist applies specific techniques during therapy that allow for collaboration between patient and therapist, the therapeutic alliance is built within the specific technique, which further enhances the effectiveness of the treatment [3]. The link between the therapeutic alliance and the effectiveness of psychotherapy has been confirmed in several meta-analyses for a range of populations and outcomes in adult patients, with moderate to large effect sizes [4–6]. However, the problem with this association is that there is great methodological diversity in terms of the measurement methods used (e.g. questionnaires, objective ratings) and the perspective from which the therapeutic alliance is measured.

There has been concern that patients suffering from Posttraumatic stress disorder (PTSD) may have difficulty forming a strong therapeutic alliance, as one of the associated problems is distrust of others, disrupted interpersonal relationships due to interpersonal traumatization or lack of social support [7]. This is particularly likely for victims of violence in childhood and adolescence [8, 9]. Furthermore, as avoidance is a core symptom of PTSD and it is also associated with difficulties in emotion regulation, trauma therapy with exposure-based elements may strain the therapeutic alliance or promote treatment dropout [10–12]. Contrary to these expectations, a meta-analysis of 34 studies by Howard et al. [13] confirmed that the therapeutic alliance was consistently rated similarly high by patients with PTSD as in other disorders, and this was consistent regardless of the type of treatment chosen. In this context, the therapeutic alliance significantly predicted PTSD symptom reduction (combined moderate effect of *r* = .34). The authors point out that there is still a need for research into possible predictors of the strength of the therapeutic alliance. However, in the studies reviewed, they were able to evidence that, for example, therapeutic competence as well as the patient’s attachment style and coping mechanisms are related to the strength of the therapeutic alliance [13].

### Therapeutic alliance in young patients

The findings described above all originate from the field of adult psychotherapy. For younger patients they do not seem to be fully transferable due to various developmental particularities. On the one hand, it is challenging to involve not only the children and adolescents but also their caregivers in the treatment process. The therapeutic alliance can be disrupted if the therapist, patient and caregiver do not agree on the goals of the therapy [14]. On the other hand, some of the young patients did not come up with the idea of going to therapy themselves, but were referred to treatment by their caregivers or other adults, which may contribute to a lower willingness to change and to bond with the therapist [15–17]. Adolescent patients in particular may disagree with their caregivers and might have different ideas about the origin of their problems or question whether therapy is necessary at all [18]. This can be problematic since successful therapy requires the active cooperation of the patient and the therapeutic alliance has been shown to be a significant predictor of this engagement during treatment [19, 20]. Therefore, a lack of therapeutic alliance may be one of the key factors contributing to dropout and poor outcomes in young patients [20, 21]. In addition, the therapeutic relationship may be influenced by the fact that, from a developmental psychology perspective, the desire for independence and self-determination is an important developmental task in adolescence - respecting and acknowledging this may facilitate consent and the setting of therapeutic goals and tasks [22]. In the most recent meta-analysis published by Karver et al. [21], a significant relationship between the therapeutic alliance and treatment outcome was found across 28 studies (small to medium effect of *r* = .19) in different clinical samples with young patients. However, the meta-analysis did not provide a consistent picture of the mechanisms underlying this association. In the studies considered there were some potential mediators examined such as participation during treatment, attendance of sessions or compliance with homework assignments [23, 24]. Nevertheless, no study has yet been able to confirm any of these mediations, partly due to lack of power and inadequate measures of alliance and treatment outcome [21]. In contrast to the adult literature, the relationship between the therapeutic alliance and treatment outcome in children and adolescents is not independent of the type of therapy: It has been shown that the therapeutic alliance plays a greater role in behavioral therapies in comparison to psychoanalytic approaches [21, 25]. There are also significant differences in the strength of the therapeutic alliance between different treatment modalities [26]. This may be because behavioral approaches usually require the active co-operation of the patient, and in therapeutic interventions such as exposure, it is crucial that the patient and therapist share the same treatment goal. However, these findings on differences between therapeutic approaches with regard to the therapeutic alliance lack validity due to the great heterogeneity of the definitions applied and measurement methods used in the studies available [21, 27].

### Therapeutic alliance and treatment outcomes in young patients with PTSD

Compared to adults, the field of therapeutic alliance between therapists and traumatized children and adolescents is understudied. However, investigating the role of alliance seems particularly necessary as traumatized young patients often have generalized dysfunctional thoughts regarding danger, betrayal or trustworthiness of others [28] which may make it difficult for them to build trust with the therapist in treatment [29, 30]. This can be an important factor in the area of interpersonal trauma, as children or young people who have experienced abuse by a caregiver often have difficulty building trust in relationships [8, 9]. For example, adult female patients who had experienced sexual abuse in their childhood were shown to have greater difficulties in establishing a therapeutic alliance than patients who did not have a history of abuse [31].

There have been some studies focusing ontherapeutic alliance in the area of PTSD in children and adolescents: In trauma-focused cognitive behavioral therapy (TF-CBT), the therapeutic working alliance has been shown to be a significant predictor of treatment outcome [29, 32]. Capaldi et al. [33] used hierarchical linear models to analyze the development of the therapeutic alliance over the course of treatment and showed that sexually abused adolescent patients with PTSD rated the therapeutic alliance significantly more positively over the course of therapy. Interestingly, the alliance ratings were even higher in the intervention group with exposure-based treatment than in the patients treated with a client-centered treatment. They also found a significant negative correlation between the therapeutic alliance and the levels of PTSD symptomatology as the number of sessions increased for both conditions. Kirsch et al. [34] showed that the therapeutic alliance of the caregivers is also important in association with therapy success in TF-CBT, where caregivers are included closely into the treatment process: the higher it was, the lower the posttraumatic stress symptoms were post treatment. Ovensted et al. [30] focused on which therapist’s behaviors promote the development of a good therapeutic working relationship with young PTSD patients. They found that techniques such as cognitive restructuring, offering support or focusing on the adolescent’s experience at the beginning of therapy were associated with improvements in the therapeutic relationship. They also showed that including trauma-related interventions (even in the early stages of therapy) did not negatively affect the therapeutic relationship.

### The present study

As described above, there is a lack of research on therapeutic alliance in younger patients with PTSD, particularly in populations with abuse-related PTSD. We therefore conducted a secondary analysis of the therapeutic alliance in the randomized controlled trial (RCT) of Developmentally adapted cognitive processing therapy (D-CPT) in adolescents and young adults with abuse-related PTSD [35]. We focused on the question of whether therapeutic alliance was associated with treatment outcome in terms of self-rated symptom reduction during D-CPT. We looked at ratings from both the patients themselves and the therapists. Based on the alliance research with adult patients with PTSD [13], different adolescent populations [21], and the existing findings on younger patients with PTSD [29, 32, 33], we stated the following hypotheses:

1. The therapeutic alliance increases with the number of sessions.
2. There is a significant negative relationship between the therapeutic alliance and self-rated PTSD symptoms over the course of D-CPT.

## Methods

### Procedure and participants

Adolescents and young adults aged 14–21 years were recruited as part of an RCT [35] on the effectiveness of D-CPT between July 2013 and June 2015 at three different locations in Germany. The ethics committees of all participating sites approved the study. Following informed consent of patients as well as caregivers, the baseline assessment was performed. Participants were then randomly assigned to either the D-CPT group or a wait-list condition with treatment advice (WL/TA). In the present analysis, we only considered the intervention group treated with D-CPT. The following inclusion criteria were set: A primary abused-related (sexually and/or physically) PTSD diagnosis according then valid DSM-IV [36] with a lowered threshold for avoidance symptoms [37]. In addition, patients were not allowed to receive pharmacological treatment unless they had been on stable medication for at least 3 weeks. Participation in the study was not possible if life-threatening suicidality or self-harming behavior had occurred (previous 6 months). In addition, patients with an IQ ≤ 75 were not included. Further exclusion criteria were: concurrent other psychotherapeutic treatments, profound developmental disorders as well as psychotic or bipolar diagnoses. Given the outpatient setting, patients with substance-related disorders or substance dependence who had been abstinent for less than 6 months were excluded.

At baseline, the PTSD-diagnosis was established by trained raters with the *Clinician Administered PTSD Scale – Child and Adolescent version* [CAPS-CA; 38] in German [IBS-KJ; 39]. The comorbidities were established using the *Structured Clinical Interview for DSM-IV Axis I* [SCID I;, 40] – in German [41]; the Borderline section from the *Structured Clinical Interview for DSM-IV Axis II* [SKID II;, 42] – in German [43]; as well as relevant sections (e.g., separation anxiety disorder, conduct disorders) for diagnoses that led to exclusion from the *Diagnostic Interview for Mental Disorders in Childhood and Adolescence* [Kinder-DIPS; 44].

### Treatment

D-CPT is a developmentally appropriate version of classical Cognitive Processing Therapy [45] that is specifically designed to meet the needs of adolescent and young adult patients with PTSD related to physical and/or sexual abuse [46]. The therapy consists of 4 different phases in which the following treatment priorities are set: commitment (phase 1), emotion regulation (phase 2), intensive D-CPT with impact statement (phase 3) and developmental tasks (phase 4). The therapy is carried out in 30 sessions of 50 min each in an high-intensity setting (30 sessions in 16 to 20 weeks, in phase 3 approximately 15 sessions in 4 weeks), with six additional joker sessions for joint caregiver sessions or crisis intervention that can be used at any point during the treatment. More detailed information can be retrieved from Matulis et al. (2014). The treatments were carried out by 14 therapists, ten licensed therapists and four therapists in training. Four therapists were male. They had an average clinical experience of 46.1 months (*SD* = 19.3). The average experience in the treatment of PTSD cases before the study was 3.3 cases (*SD* = 5.6). The therapists were trained in D-CPT in a three-day workshop with subsequent refreshing sessions. In addition, fortnightly case consultations were conducted by telephone by licensed supervisors.

### Measures

#### Helping alliance questionnaire (HAQ)

To assess the therapeutic alliance, the *Helping Alliance Questionnaire* (HAQ) [47, 48] was completed by both patients and therapists after each session. For the present study, the adapted 11-item version [49] was applied. Patients and therapists rated their satisfaction with the alliance (e.g. “I think my therapist is helping me”) and the success of the therapy (e.g. “I feel better lately”) on a six-point Likert scale ranging from 1 (“very unapplicable”) to 6 (“very applicable”). Higher scores indicate a stronger therapeutic alliance.

The instrument was validated by Nübling et al. [49] in three clinical samples (*N* = 4626) in different treatment settings with both behavioral and psychodynamic therapeutic orientations. The validity as well as the very good internal reliability could be confirmed in all samples, which shows that the HAQ, despite its psychodynamic origin, can be used in different clinical settings. In the present sample, the total sum scores had a good to excellent internal consistency of *α = .*899 (HAQ_therapists_) and *α = .*923 (HAQ_patients_).

#### Clinician-administered PTSD scale for children and adolescents (CAPS-CA)

The *Clinician-Administered PTSD Scale for Children and Adolescents* [38] is a commonly used clinically structured interview for diagnosing PTSD according to DSM-IV. At study intake, we used the German version by Steil & Füchsel (2006). Trained, independent raters evaluated the severity of symptoms on a scale ranging from 0 (“never/no problem”) to 4 (“most of the time/extreme”), with higher scores indicating more severe symptoms. The maximum total sum score possible is 136. Cronbach’s alpha for the total sum score was *α* = 0.875. Subscales had acceptable to good reliability (intrusion *α* = 0.785; avoidance *α* = 0.626; hyperarousal *α* = 0.618).

#### University of California at Los Angeles PTSD reaction index for DSM-IV (revision 1; UCLA-PTSD-RI)

The German version [50] of the *University of California at Los Angeles PTSD Reaction Index for DSM-IV of the UCLA-PTSD-RI* [51] was applied to measure self-reported PTSD symptom severity after each session. The questionnaire consists of three parts: A brief screening of PTSD events, a systematic assessment of the event according to the A-criterion of the DSM-IV and a symptom scale. On the symptom scale, 20 items differentiate between three different subscales according to the symptom classes: intrusion, avoidance and hyperarousal. Answers were given on a 5-point Likert Scale ranging from 0 (*none)* to 4 (*most of the time*). The maximum total sum score is 68. Again, higher scores indicated a greater symptom severity. In our sample, the sum score had a good reliability *α* = 0.841, whereas the internal consistency for the subscales were acceptable (intrusion *α* = 0.785; avoidance *α* = 0.615; hyperarousal *α* = 0.627).

### Data analysis

For each treatment case, we determined an average HAQ score over the entire treatment process (for HAQ_patients_ as well as HAQ_therapists_). Alliance measures have, as here, a nested structure [4]. Individual session numbers (level 1) are nested within different treatment cases (level 2). We therefore decided to assess the variance at the respective levels and evaluated the available data using multilevel modelling (MLM). In contrast to repeated-measures analysis of variance (ANOVA), MLM has the additional advantage that missing values can be dealt with more flexibly, and all observations of the therapeutic alliance can be taken into account from all treatment cases who have at least one observation [52, 53]. Of course, one can argue that there is a third level as treatment cases are nested in therapists. However, the number of cases per therapist and the total number of therapists were not sufficient to include therapist-level effects in the models. While therapist factors could influence the development of the therapeutic alliance and its relationship with treatment outcomes, the available data did not allow for precise modeling of these effects.

We built each model in a stepwise approach: first, we conducted a null model with a random intercept to determine individual differences in therapeutic alliance at baseline. We then calculated the interclass correlation (ICC). As this was significantly deviated from 0, we assumed that there were grouping effects based on the nested structure, which allowed to continue within the MLM framework. We added the session number as a fixed predictor (random slope x random intercept model) to determine whether therapeutic alliance improves with increasing session numbers (hypotheses 1). We set up the models with both the HAQ_patients_ and the HAQ_therapists_.

For hypothesis two, we conducted a null model with a random intercept to determine individual differences in symptom severity at baseline. We then calculated the interclass correlation (ICC). Following this, we added the session number as a fixed predictor. In the last step, we added an interaction effect between the session number and HAQ as a predictor to test the relationship between therapeutic alliance and treatment outcome during treatment (hypothesis 2). When setting up the models we allowed free covariation of the random effects. We tested their significance with likelihood ratio tests. Model fit was estimated by comparing the random variance to a model without random variance. The significance of the fixed effects was calculated using a standard estimator of degrees of freedom.

For all tests, we applied a common alpha level of 0.05. Statistical analyses were proceeded with R-Studio and IBM SPSS Statistics 27 for Windows.

## Results

After study intake, 44 patients were randomized to the D-CPT group. Only treatment cases with at least one therapeutic alliance observation were included in our analyses. As two individuals were never able to start therapy for organizational reasons, two patients were false randomizations and one person dropped-out before rating therapeutic alliance, the present sample consisted of *n* = 39 treatment cases. Demographic information and baseline PTSD symptom severity of the sample are shown in Table 1.

**Table 1:** Demographics and symptom severity at study intake

	Study Population
	D-CPT Group (*n* = 39)
Age, mean (95% CL)	18.2 (14.3–21.9)
Female, No. (%)	36 (92)
Posttraumatic Stress Symptom Score	
CAPS-CA, *M (SD)*	67.97 (23.05)
UCLA, *M (SD)*	42.28 (10.51)
Comorbid DSM-IV disorders, No. (%)	
0	9 (23)
1 or 2	21 (53)
≥ 3	9 (23)
Most frequent DSM-IV disorders, No. (%)	
Mood disorders	20 (51)
Anxiety disorders	14 (36)
Nicotine dependence	10 (26)
Borderline personality disorder	4 (10)
Trauma, No. (%)	
Only physical	8 (21)
Only sexual	6 (15)
Both	25 (64)

Note: *CAPS-CA* = Clinician-Administered PTSD Scale for Children and Adolescents for DSM-IV, *D-CPT* = Developmentally Adapted Cognitive Processing Therapy, *PTSD* = posttraumatic stress disorder, *UCLA* = University of California at Los Angeles Posttraumatic Stress Disorder Reaction Index

### Descriptive data on therapeutic alliance

For several reasons, the number of observations collected for therapeutic alliance (as well as PTSD treatment severity) varied between the treatment cases analyzed over the course of therapy e.g. dropouts during the trial, to optional sessions for crisis intervention or joint sessions with the caregiver or missing entries in the questionnaires). Although we were very concerned to obtain an assessment of the development of symptoms and the therapeutic alliance from the patients as well as therapists after each session, compliance varied from subject to subject. The answering rate of the patients was 97%, whereas the answering rate of the therapists was 95%.

All in all, we included *n* = 679 observations for HAQpatients and *n* = 664 observations for HAQ therapists. The mean therapeutic alliance rated by patients was M = 55.58 (SD = 8.94) with a range from 11 to 66, the mean therapeutic alliance rated by therapists was M = 50.02 (SD = 6.97) with a range from 27 to 66. For an overview over the course of treatment, Table 2 shows the number of observations, mean values and standard deviations for therapeutic alliance ratings, separated according to therapy phases, as well as the self-reported symptom severity of the patients.

**Table 2:** Means of therapeutic alliance and symptom severity in different treatment phases

Treatment phase	Phase 1		Phase 2		Phase 3		Phase 4	
	*n*	M (SD)	*n*	M (SD)	*n*	M (SD)	*n*	M (SD)
HAQ_patients_	157	51.36 (8.88)	156	55.76 (6.30)	207	56.80 (8.04)	106	60.89 (7.19)
HAQ_therapists_	157	45.70 (6.21)	154	49.72 (5.19)	202	52.09 (6.21)	101	54.54 (6.94)
UCLA	164	38.40 (13.49)	158	34.93 (14.14)	211	29.63 (14.38)	109	24.74 (13.99)

Note: *HAQ* = Helping Alliance Questionnaire, *UCLA* = University of California at Los Angeles Posttraumatic Stress Disorder Reaction Index, *n* = number of observations; differences in *n* are due to missing entries and different lengths of treatment phases

At the end of treatment, 13 patients (34%) of the sample studied achieved clinically significant improvements (measured as a change of 2 SD below baseline). We also calculated the Pearson product-moment correlations for the symptom severity and therapeutic alliance measures, which are shown in Table 3.

**Table 3:** Correlations between symptom severity and therapeutic alliance measures

	HAQ_patients_	HAQ_therapists_	UCLA
HAQ_patients_	1	0.512**	− 0.460**
HAQ_therapists_	0.512**	1	− 0.363**
UCLA	− 0.460**	− 0.363**	1

Note: *HAQ* = Helping Alliance Questionnaire, *UCLA* = University of California at Los Angeles Posttraumatic Stress Disorder Reaction Index; * *p* < .05, ** *p* < .01

Based on the described correlation between HAQ_patients_ and the HAQ_therapists_, we tested whether the patient and therapist HAQ ratings differed significantly per session. As the differences were not normally distributed (Shapiro-Wilk tests *p* < .000), we applied the non-parametric Wilcoxon signed-rank test. It showed that the HAQ_patients_ were significantly higher than the HAQ_therapists_ per session (*p* < .000).

### Therapeutic alliance during treatment

In order to analyze the first hypothesis concerning the course of the therapeutic alliance during treatment, models were set up gradually for HAQ_patients_ and HAQ _therapists_. We began with HAQ_patients_: the null modell with an ICC of 0.70 indicated grouping effects. The intercept (fixed effect) offers an estimation of the baseline scores. The estimate for this fixed effect was 54.58 with *SE* = 1.21 and *t* = 45.13 (*DF* = 38.45), *p* < .001. Following this, the comparison between the fixed slope and random slope model revealed that incorporating a fixed slope enhanced the model (*χ2* (2) = 98.64, *p* < .001). The ultimate random intercept with random slope model, variations specific to each session, compared to this baseline term, were estimated. The random effects delineate the approximate distinctions among patients (random intercept) and the patient-specific alterations in therapeutic alliance for each session (random slope). The variance between patients concerning therapeutic alliance was 71.32, the patient-specific alterations was 0.05 with a residual variability of 13.19 (n_*observations*_ = 679, *n*_*patients*_ = 39). Fixed effects showed a significant increase in therapeutic alliance (HAQ_*patients*_) with increasing session duration (Table 4).

**Table 4:** Fixed effects from multilevel modeling of therapeutic alliance during treatment

	Estimate (SE)	t (DF)	*p*
HAQ_patients_			
Intercept	50.61 (1.39)	36.51 (34.41)	< 0.000 ***
Session	0.29 (0.04)	7.08 (30.11)	< 0.000 ***
HAQ_therapists_			
Intercept	45.44 (0.82)	55.60 (36.96)	< 0.000 ***
Session	0.27 (0.08)	6.94 (32.69)	< 0.000 ***

Note: *HAQ*_patients_ = Helping Alliance Questionnaire answered by patients; *HAQ*_therapists_= Helping Alliance Questionnaire answered by therapists; * *p* < .05, ** *p* < .01, ***p < .000

Simultaneously, we proceeded with HAQ_therapists_: the null model, characterized by an ICC of 0.52, demonstrated grouping effects. The fixed effect estimate was 49.26 (*SE* = 0.84) and *t* = 58.54 (*DF* = 38.10), *p* < .001. The subsequent step again favored a model with a random slope over a fixed slope (χ2 (2) = 94.62, *p* < .001). The variance attributed to therapeutic alliance between patients was 22.98, patient-specific alterations were 0.04 and residual variability was 13.39 (*n*_*o*bservations_ = 664, *n*_patients_ = 39). The fixed effects indicated a significant elevation in therapeutic alliance (HAQ_therapists_) with an increase in session duration (refer to Table 4).

### Relationship between symptom reduction and therapeutic alliance during treatment

**Fig. 1:**
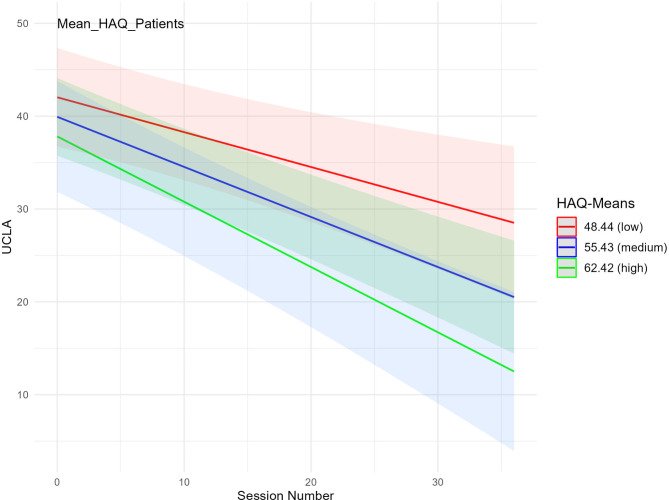
Interaction between therapeutic alliance (*HAQ*_*patients*_) and treatment course (*session number*) to describe symptom severity (*UCLA*). *HAQ*_patients_ = Helping Alliance Questionnaire answered by patients, *UCLA* = University of California at Los Angeles Posttraumatic Stress Disorder Reaction Index

To answer the second hypothesis regarding the relationship between symptom severity reduction and the therapeutic alliance over the course of treatment we set up a model of self-rated symptom severity predicted by a cross-level interaction between therapeutic alliance and the number of sessions. Again, regarding therapeutic alliance as cross-level predictor, a distinction was made between HAQ_patients_ and HAQ_therapists_, and 2 equivalent models were calculated for each. We started with HAQ_patients_: The model with the cross-level interaction was superior to the model with the random intercept and random slope (*χ2* (2) = 8.66, *p* = .013). The estimates for the random effects in this model were 161.34 for between patient variance and 0.20 for patient-specific session variance with a residual variability of 25.15 (*n*_*o*bservations_ = 698, *n*_patients_ = 39). Fixed effects are shown in Table 5 and indicated significant cross-level interaction: patients with higher therapeutic alliance had a significantly greater reduction in symptom severity over the course of treatment than patients with lower therapeutic alliance scores. This interaction is illustrated in Fig. 1. As an example, the course of symptoms using UCLA-PTSD-RI over different sessions for different treatment cases is shown. Treatment cases with a higher HAQ_patient_ (shown here in green) had a greater reduction in symptoms than treatment cases with a lower mean HAQ _patient_ (shown here in red).

It should be noted that the relationship could of course be reversed and that patients with lower symptom severity generally perceived a better therapeutic alliance over the course of treatment. We therefore also ran the model inversely, with HAQ_patients_ as the dependent variable and symptom severity as a cross-level predictor. This showed that there was no significant interaction effect between PTSD symptom severity and the session number in predicting therapeutic alliance (as rated by the patient) (all fixed effects *p* > .05).

Concurrently, we continued with HAQ_therapists_ as cross-level predictor for symptom severity: The model incorporating the cross-level interaction could not describe the data structure significantly better than the model with the random intercept and random slop (*χ2* (2) = 2.41, *p* = .299). Because we suspected that the effects might be very small, and due to the importance of the interaction of therapeutic alliance and treatment duration on symptom reduction as described in the theoretical background, we decided to consider the more complex model anyway. The estimates for the random effects in this model were 172.17 for between-patient variance. Patient-specific variance between session was 0.22, residual variability was 25.13 (*n*_observations_ = 698, *n*_patients_ = 39). Fixed effects can also be retrieved from Table 5. In contrast to the model with HAQ rated by patients, there was no significant cross-level interaction. This suggests that there is no significant interaction between therapist-rated therapeutic alliance and treatment duration in terms of symptom reduction.

**Table 5:** Fixed effects from multilevel modeling of the relationship between therapeutic alliance and symptom severity during treatment

	Estimate (SE)	t (DF)	*p*
UCLA			
Intercept	64.72 (15.31)	4.23 (41.57)	< 0.000 ***
Session	0.77 (0.61)	1.26 (39.70)	0.215
HAQ_patients_	-0.45 (0.28)	-1.62 (41.23)	0.114
HAQ_patients_ x session	-0.02 (0.01)	-2.16 (38.73)	0.037 *
UCLA			
Intercept	45.52 (19.97)	2.48 (39.51)	0.018 *
Session	0.63 (0.82)	0.76 (37.31)	0.448
HAQ_therapists_	-0.19 (0.40)	-0.47 (39.34)	0.644
HAQ_therapists_ x session	-0.02 (0.02)	-1.43 (36.58)	0.161

Note: *UCLA* = University of California at Los Angeles Posttraumatic Stress Disorder Reaction Index; *HAQ*_patients_ = Helping Alliance Questionnaire answered by patients; *HAQ*_therapists_= Helping Alliance Questionnaire answered by therapists; * *p* < .05, *** *p* < .000

## Discussion

The aim of the present study was to answer the question if the therapeutic alliance is associated with treatment outcome during the course of D-CPT in adolescent PTSD patients following sexual and/or physical abuse. To meet the need for data on young patient populations, both the PTSD symptom severity and the alliance ratings of the therapists and patients were analyzed on the basis of weekly measurements after each session.

### Course therapeutic alliance during treatment

We showed that the therapeutic alliance improved significantly with increasing number of sessions, supporting the first hypothesis. This in agreement with the findings in adult PTSD populations [13] and the study of Capaldi et al. [33], which also found an improvement in the therapeutic alliance during the course of treatment in a sample of adolescents with sexually abuse related PTSD using MLM. In our study, this result was independent of whether the therapeutic relationship was assessed by the patients or by the therapists. In both cases there was a significant improvement in therapeutic alliance with increasing number of sessions. However, although we found a strong correlation between patients’ and therapists’ ratings, there was a significant difference between patients’ and therapists’ ratings per session with patients perceiving the therapeutic alliance to be significantly better than their therapists. These results indicate that there is a connection between the patient’s and therapist’s perception of the therapeutic alliance across all measurement points, but that the therapist’s assessment of the therapeutic alliance per session is on average lower than that of the patient. The findings in the literature are not unambiguous about the agreement of judgement concerning therapeutic alliance. In the field of PTSD, Therapist-rated alliance was consistently lower than client-rated alliance [13]. In the context of substance-abuse disorders in adult patients, a meta-analysis by Shick Tyron et al. [54] also found only a medium correlation between therapist and patient judgement; overall, patients rated the therapeutic alliance significantly more positively, like in our study. One possible explanation is that the two sides differ in terms of experiences. Research has revealed that the evaluations of the therapeutic alliance can be influenced differently by patients and their therapists, depending on the processes or emotions experienced in the same sessions [55]. Additionally, while therapists can draw on their experience with other patients, patients may not have had any previous therapy and can only draw on their experiences with other professionals in the health care sector or on previous experiences of friends or acquaintances in psychotherapy [54]. In contrast to that, the meta-analysis by Flückiger et al. [4] of studies with adult clinical populations found no significant differences between therapists’ and patients’ perspectives among a range of disorders. This was opposite to previous meta-analytic approaches that also tended to show lower average therapist ratings in different clinical samples [5, 6].

### Relationship between therapeutic alliance and treatment outcome during D-CPT

With regard to the second hypothesis, it was shown that patients with a higher therapeutic alliance (rated by patients) achieved a greater reduction in PTSD symptoms over the course of treatment than patients with a lower therapeutic alliance. This shows that in D-CPT there is also a significant relationship between the therapeutic alliance and the treatment success over the course of sessions. This is congruent with findings from research with adult patients with PTSD [13] and other adult clinical samples [4, 5]. Similar results were also found in studies with children and adolescents with various disorders [21] as well as specific studies with young PTSD populations [29, 32, 33]. However, the negative correlation between therapeutic alliance and symptom reduction over the course of therapy is only significant for the judgement of the patients themselves. In this case, the patients’ evaluation of the therapeutic alliance seems to be more important for treatment outcome than the therapists’ judgement. This is opposing to a study by Baldwin et al. [56] that separated the patient’s and therapist’s contributions to the alliance in adult psychotherapy. They found that the therapist’s ratings of the alliance significantly predicted outcomes, whereas the patient’s contribution did not show a significant effect. This so-called therapist effect was replicated later on [57]. However, findings from adult alliance research cannot fully be transferred to adolescent samples [27]. A recent study by Ovenstad et al. [30] on traumatized adolescents with PTSD comparing different raters perspectives showed that only patient ratings could predict treatment outcome in terms of symptom reduction.

Of course, one can state the question whether an improvement in symptom reduction might not cause an improvement in therapeutic alliance in the first place, so we reversed the model. However, we found that symptom severity across sessions was not a significant predictor of therapeutic alliance, suggesting that therapeutic alliance is more likely to lead to symptom reduction across sessions. This has also been found in other adolescent samples with sexual abuse related PTSD, where an association between therapeutic alliance and treatment success over the duration of therapy was found, but not vice-versa [33].

### Strengths of the study

This study is one of the few focusing on young patients with abuse related PTSD. These patients often face problems forming trusting relationships, particularly when having experienced interpersonal violence [8, 9, 29]. In addition, common dysfunctional beliefs about danger and betrayal or the PTSD core symptom of avoidance can complicate the establishment of the therapeutic alliance [12, 28]. In addition, adolescents are often referred to therapy by third parties [17, 18], or are in a developmental phase in which freedom and self-determination are most important [22]. The present study therefore provides needed findings on a specific sample that has not yet been sufficiently studied in psychotherapy research regarding alliance.

We were also able to use progression estimates based on weekly measurements. This has the advantage that we did not simply rely on measurement points at the beginning, middle and end of the treatment, but included the full dynamics of the process over the treatment sessions in our calculations.

As the therapeutic alliance is a dyadic concept [3], a further advantage of our study is that we assessed both the patients’ and the therapists’ perceptions. In several studies, only the ratings from patients are considered. This creates a bias since the same individual is then responsible for evaluating both the treatment’s success and the therapeutic alliance [5, 13]. In addition, the predictors of therapeutic alliance differ between these two perspectives. In adult PTSD patients, the only consistently found predictor is attachment style [13]. Others, such as overt emotional expression during sessions or a patient’s coping style [58, 59], have been discussed, but there is still insufficient evidence [13]. At the therapist level, higher competence is associated with higher alliance in therapy [58]. However, a previous study from our D-CPT trial showed that therapeutic adherence and competence did not predict treatment success, neither post treatment nor at follow-up [60]. This study also controlled for patient-rated therapeutic alliance on this association. It was shown that there was no significant correlation between the therapeutic alliance and a therapist’s adherence or competence. However, in contrast to adherence and competence, higher patient-rated alliance was associated with lower symptom severity at 12 months post treatment.

### Limitations and further research

The results are constrained by several factors. Firstly, we only applied self-report measures. Although we have already considered two possible sources for measuring the therapeutic alliance, the perspective of an objective observer is missing. In contrast to TF-CBT, D-CPT does not regularly include joint sessions or sessions with caregivers as it was developed for adolescents and young adults – therefore, the perspective of caregivers on therapeutic alliance is not missing. Future studies should include independent ratings of therapeutic alliance. However, the meta-analysis on adult psychotherapy conducted by Flückiger et al. [4] revealed a tendency wherein the effects observed by external assessors were slightly less pronounced when compared to the correlation between client-rated alliance and treatment outcomes.

Secondly, the D-CPT manual explicitly incorporates components focused on fostering the therapeutic alliance between the therapist and the client at the beginning of the treatment process [61]. Consequently, our study cannot provide conclusive statements about protocols that lack these initial elements. It is recommended that future research includes a comparison between D-CPT and other PTSD treatments for young patients, for a more comprehensive understanding.

Another limitation is that our sample of therapists may not be fully representative. In our multicentre trial, all clinical sites had a high level of expertise in the treatment of PTSD associated with sexual and/or physical abuse. The average levels of adherence and competence were markedly high and tended to fall within the maximum range of the scales [60]. This is plausible as this trial was designed to focus on treatment evaluation of D-CPT and therefore needed to maintain high levels of adherence and competence through structured therapist trainings to ensure internal validity. However, there is evidence that the level of experience and competence is positively associated with the level of therapeutic alliance [13, 58]. This could not be confirmed for our trial, as there was no significant association between therapeutic competence and the levels of patient rated therapeutic alliance [60]. The relationship between therapeutic competence, therapeutic alliance and treatment outcomes therefore needs investigation in future dissemination trials, where a wider range of therapeutic expertise can be anticipated.

Moreover, we did not perform a separate power analysis for the secondary analyses, as it was difficult to estimate the expected effect sizes based on the available data on samples with abuse related PTSD in children and adolescents. Although a power analysis was performed for the primary RCT, we acknowledge that future studies should include power analyses for secondary outcomes to strengthen the interpretability of the results. In terms of treatment quality, it should be added that 34% of patients achieved a clinically significant change in the severity of PTSD symptoms (2 SD below baseline). However, 20 patients were already below this threshold before the start of treatment and were therefore unable to achieve a clinically significant change. The results discussed in this paper therefore do not exclusively refer to treatment successes in the clinically significant range.

Due to the small sample size, including PTSD symptom severity at baseline as a predictor in the models compromised the stability of the analyses, leading to unreliable estimates. The inclusion of baseline symptom severity did not significantly improve the model fit (*χ²* = 2.58, *p* = .108; *BIC* (4555.3 vs. 4495.4)). However, to address this concern, we also conducted an inverted model in which we predicted therapeutic alliance based on symptom severity. The results indicated that symptom severity did not significantly predict the therapeutic alliance, suggesting that the effect of symptom severity on the alliance-outcome relationship is unlikely. In future studies with larger sample sizes, including baseline PTSD symptom severity could provide valuable insights. However, existing research in adult PTSD populations also suggests that the alliance-outcome link is not significantly influenced by symptom severity at the beginning of treatment [13].

### Clinical implications

Contrary to many concerns, young patients with a history of sexual and/or physical abuse can develop stable therapeutic alliances during PTSD treatment. The therapeutic alliance is an important factor in D-CPT, as it contributes to the reduction of symptom severity over the course of the treatment. However, in this context, the patients’ rating is more important than that of the therapists. Therapists should therefore pay particular attention to building a stable alliance and ask their patients for their perception. Routine outcome monitoring is a powerful tool that can increase the likelihood of detecting changes in the therapeutic alliance early on, especially in cases where patient-perceived alliance is low. For example, patients could answer the HAQ [47, 48] at regular intervals. As the questionnaire used in this study consisted only of 11 items [49], it is an economic way of keeping track of the therapeutic alliance during treatment. This monitoring should be discussed openly and transparently with patients, fostering a collaborative relationship where both therapist and patient are attuned to and engaged in the ongoing process of alliance-building. If the alliance is deteriorating, they can first check whether this is in line with the patients’ perception. If this is the case, and the alliance results also show that, therapists should not overestimate their own perception of the therapeutic relationship. If a therapist feels that the therapeutic alliance is on a low level, the therapist can use targeted techniques to help improve the therapeutic relationship It has been shown that interventions like cognitive restructuring and offering support are associated with higher levels of therapeutic alliance in adolescent PTSD patients [62].

## Conclusions

This study of adolescent patients with PTSD following sexual and/or physical abuse investigated whether there was a connection between the therapeutic alliance and symptom reduction over the course of D-CPT analyzing weekly measures with MLM. We found that the therapeutic alliance increased significantly with ongoing session duration. Therapists’ and patients’ ratings of the therapeutic alliance were strongly correlated. However, they differed significantly at the session level. Patients perceived the alliance to be higher than their therapists. Patients with a better therapeutic alliance showed a greater reduction in self-rated PTSD symptoms over the course of therapy. However, this relationship between therapeutic alliance and treatment success in D-CPT was only found for the patient ratings, suggesting that these were more important for PTSD symptom reduction in the present sample. Therapists should therefore focus on building a strong alliance and explicitly ask for their patients’ judgement. Future studies should concentrate on independent alliance ratings, symptom severity at the beginning of the treatment and larger sample sizes further examine the alliance-outcome link.

## Data Availability

The datasets and materials produced or analyzed in the present study can be obtained from the corresponding author upon a reasonable request.

## Abbreviations

ANOVA: Analysis of variance
CAPS-CA: Clinician-Administered PTSD Scale for Children and Adolescents
CPT: Cognitive processing therapy
D-CPT: Developmentally adapted cognitive processing therapy
DF: Degrees of freedom
HAQ: Helping Alliance Questionnaire
ICC: Interclass correlation
M: Mean
MLM: Multilevel modeling
PTSD: Posttraumatic stress disorder
RCT: Randomized controlled trial
SCID I: Structured Clinical Interview for DSM-IV Axis I
SCID II: Structured Clinical Interview for DSM-IV Axis II
SD: Standard deviation
TF-CBT: Trauma-focused cognitive behavioral therapy
UCLA-PTSD-RI: University of California at Los Angeles PTSD Reaction Index for DSM-IV, (Revision 1)
WL/TA: Wait-list condition with treatment advice
